# Effects of *Lactobacillus plantarum* (L) and molasses (M) on nutrient composition, aerobic stability, and microflora of alfalfa silage in sandy grasslands

**DOI:** 10.3389/fmicb.2024.1358085

**Published:** 2024-04-23

**Authors:** Wen Peng, Liyuan Zhang, Manlin Wei, Baiyila Wu, Ming Xiao, Runze Zhang, Ji Ju, Chenyang Dong, Liu Du, Yongjie Zheng, Meili Bao, Hailin Bao, Xiaoping Bao

**Affiliations:** ^1^College of Animal Science and Technology, Inner Mongolia Minzu University, Tongliao, China; ^2^Horqin Left Wing Rear Banner National Vocational and Technical School, Tongliao, China; ^3^DongYing AustAsia Modern Dairy Farm Co., Ltd, Dongying, China

**Keywords:** alfalfa silage, *Lactobacillus plantarum*, molasses, fermentation quality, aerobic stability, microflora

## Abstract

The objective of this experiment was to investigate the effects of *Lactobacillus plantarum* and molasses on the nutrient composition, fermentation quality, bacterial count, aerobic stability, and microflora of alfalfa silage in sandy grasslands. The experimental treatments included control (CK), 10^6^ CFU/g *Lactobacillus plantarum* (L), 5% molasses (M), and 10^6^ CFU/g *Lactobacillus plantarum* + 5% molasses (LM). The nutrient composition, fermentation quality, bacterial count, aerobic stability, and microflora were determined after 14 days and 56 days of ensiling, respectively. The results showed that the addition of L, M, and LM reduced dry matter loss (DM), neutral detergent fiber (NDF), and acid detergent fiber (ADF) content, and increased water-soluble carbohydrates (WSC) and ether extract (EE) content, compared to the CK group. Meanwhile, more lactic acid (LA) and accelerated fermentation were observed, causing the pH value to drop below 4.5 in the L, M, and LM groups after 56 days of ensiling. The addition of L, M, and LM promoted lactic acid bacteria (LAB), and inhibited yeast. The addition of L significantly increased the content of acetic acid (AA). In terms of microflora, the addition of L, M, and LM made *Firmicutes* become the dominant bacterial phylum earlier, while *Lactobacillus, Weissella*, and *Pediococcus* had a higher abundance. According to the result of Pearson's correlation, there is a very significant negative correlation between pH value and *Lactobacillus* (*P* < 0.01) and a very significant positive correlation between pH value and *Lactococcus, Enterobacter, Enterococcus*, and *Leuconostoc* (*P* < 0.01), which may be inhibited by *Lactobacillus* under the decreased pH value. The results of the prediction of microbial genes indicated that the addition of M could enhance the carbohydrate metabolism and membrane transport metabolism, which may contribute to LA production by LAB metabolism. In general, L, M and LM all improved the fermentation quality and reduced the loss of nutrients to varying degrees, but considering the fermentation quality, the overall effects of M and LM were better than L. M and LM are recommended to be used as silage additives in the process of alfalfa silage in sandy grasslands to improve the quality.

## 1 Introduction

The Horqin grassland in the Inner Mongolia Autonomous Region of China has a temperate continental climate. Over the years, affected by climate change and grazing, the land in Kailu County, Tongliao City, has become sandy and salinized, limiting the development of cultivation, and thus changed from traditional agricultural areas to semi-rural and semi-pastoral areas. In recent years, with the implementation of grassland reclamation and sand fixation, the ecology of sandy grasslands has improved. In some areas of Kailu County, the soil nutrient content was increased by covering black soil based on the original sandy land to plant alfalfa (*Medicago sativa L*.) which could further improve soil condition through the nitrogen-fixing effect. And, on the other hand, alfalfa is used to feed livestock and to be sold as feed products.

As we know, alfalfa is a perennial herbaceous plant of the Medicago legume family, which is used to produce silage in many places and has a high nutritional value. Compared to other legume crops, alfalfa is not only richer in lysine, leucine, methionine, cysteine, and other essential amino acids and vitamins, but also contains phosphorus, calcium, potassium, sodium, chlorine, sulfur, magnesium, copper, manganese, iron, cobalt, boron, molybdenum, and other minerals required by animals (Suwignyo et al., 2020, 2023; Hidosa and Biru, 2021), and is known as “the king of pasture grass” (Suttie, 2012). Alfalfa has a strong tolerance to drought and cold and has a fast tillering and growth rate. Although alfalfa does not require high soil conditions, the poor water retention capacity of sandy areas can limit plant growth and nutritional value. Thus, research on the nutrient composition and silage quality of alfalfa grown in sandy grasslands is beneficial to the utilization of alfalfa forage in sandy grasslands.

Hay is one kind of product of sandy grassland alfalfa. However, because the first harvest of alfalfa usually occurs in June and July in the north, alfalfa hay is susceptible to mold and develops deterioration due to the rainy season, and the humid climate. In comparison, silage is a safer and more effective preservation method, because silage has a high density per unit volume and there is no leaf loss as in the drying process. Studies have shown that alfalfa silage, especially when added with appropriate additives or inoculants, can improve aerobic stability as well as the quality and nutritive value of silages (Besharati et al., 2020b, 2021b). Currently, alfalfa baled silage is widely used in the local area. Baled silage is a very practical feed processing method in semi-rural and semi-pastoral areas. Not only because of its small fixed investment, convenient preservation, and transportation but also because of its high nutritional value. The crude protein content of high-quality alfalfa silage can reach more than 18%, and the rumen degradable protein (RDP) and the organic acid content are also improved, which can provide more nutrients available to ruminants (Brito and Broderick, 2006; Coblentz et al., 2016). Feeding with alfalfa silage instead of alfalfa hay of similar quality can improve the percentage of milk fat and the percentage of milk protein of dairy cows (Calberry et al., 2003).

However, the disadvantage of alfalfa silage is its low content of fermentable carbohydrates and the high content of crude protein in the raw material, which has a strong buffer effect on the pH value during ensiling and makes it difficult to ensile separately. Especially for baled silage, since the degree of compaction of baled silage is often not as good as silo and storage silage (Broderick and Muck, 2009), it is easy to deteriorate and cause nutrient loss or animal poisoning (Driehuis et al., 2018; Penagos-Tabares et al., 2022). Adding fermentable carbohydrates to silage seems to be particularly effective for silage feed because they provide a source of sugar for LAB and are consumed in LA production (Besharati et al., 2021a; Ke et al., 2023). Molasses contains a large amount of WSC, which can provide substrates for the growth of lactic acid bacteria to promote fermentation (Xie et al., 2021; Gül, 2023). Study has shown that adding molasses to alfalfa silage could improve silage quality, gas production and in vitro DM digestibility (Besharati, 2018). When 3%-6% molasses is added, it provides WSC that can meet the energy needs of LAB and is converted to organic acids (especially LA) with high efficiency (Fahmi et al., 2019). Thus, the pH value can be rapidly reduced to inhibit the proliferation of harmful microorganisms, which could bring a reduced loss of nutrients. Moreover, molasses can also increase the synthesis of microbial proteins and improve the nutritional quality of silage (Chen et al., 2020). Besides, molasses can also improve the palatability of alfalfa silage to animals (Luo et al., 2021). Rapid initial acidification is the key to controlling the growth of competing enterobacteria, clostridium, yeasts, and molds, as well as the loss of nutrients (Khan et al., 2023). When the total population and type of natural LAB are not sufficient for the rapid initial acidification, LAB inoculants are usually needed. LAB inoculants have been proved to promote feed acidification and have been widely used in various silage production to improve the silage quality (Jia et al., 2019; Yang et al., 2020; Mu et al., 2021). Meanwhile, the bacteriocins produced by LAB also inhibit the growth of undesirable microorganisms, such as yeast and mold, to prevent deterioration of the silage (Ren et al., 2020; Yang et al., 2020; Zhang et al., 2021). When LAB and molasses are both added to the silage, there is usually a synergistic effect. Because LAB directly supplements the bacteria, while molasses provides soluble carbohydrates for LAB, and it is much more efficient than adding either of the two additives separately (Ni et al., 2017; Li Y. et al., 2022). Based on the results of previous studies, it is meaningful to investigate the addition of LAB and molasses in alfalfa ensiling in sandy grasslands.

Therefore, the objective of this experiment was to investigate the effects of *Lactobacillus plantarum* (L) and molasses (M) on the composition of nutrients, the quality of fermentation, bacterial count, the aerobic stability, and the microflora of alfalfa silage in sandy grasslands, to provide a reference for the feed processing of alfalfa silage in sandy grasslands.

## 2 Materials and methods

### 2.1 Silage preparation

The alfalfa used for silage was harvested in June 2021 in Kailu County, Tongliao City, Inner Mongolia Autonomous Region (latitude 43°9′-44°10′ N, longitude 120°25′-121°52 E) and the alfalfa samples were cut to 1–2 cm with a forage crusher. The lyophilized powder of *L. plantarum* (10^11^CFU/g) was purchased from Beijing Baiobowei Biotechnology Co. Ltd and molasses (42%-50% sugar, Brix ≥ 60) was purchased from Weifang Fengguan Biotechnology Co. Ltd.

A single-factor experimental design was adopted in the experiment, and four treatment groups were set up: control (CK), *Lactobacillus plantarum* (L), molasses (M), and *Lactobacillus plantarum* + molasses (LM). In groups L, M, and LM, 10^6^ CFU/g *Lactobacillus plantarum* lyophilized powder, 5% molasses, 10^6^ CFU/g *Lactobacillus plantarum* + 5% molasses were dissolved in 10 mL of sterile distilled water and evenly mixed in raw materials, respectively. In the CK group, 10 mL sterile distilled water was added and evenly mixed. The final mixes of each group (300 g) were packed into a special polyethylene plastic silage bag (27 cm × 39 cm, thickness 0.24 mm), sealed with a vacuum sealer, and fermented at a temperature of 23–25 °C away from light. Triplicate silages were prepared for each group, and samples were collected and analyzed after 14 days and 56 days, respectively.

### 2.2 Nutrient composition and fermentation quality analysis

Alfalfa silage was sampled and dried in a constant temperature drying oven (DHG-9240A, Shanghai, China) at 65°C for ~48 h to a constant weight to determine the dry matter (DM) (Zhang et al., 2016). The dried material samples were smashed and passed through a 1.0 mm sieve for nutrient analysis. The contents of crude protein (CP), water-soluble carbohydrate (WSC), and ether extract (EE) were determined using the Kjeldahl method (AOAC, 2000), anthrone colorimetry (Wu and Nishino, 2016), and the Soxhlet extraction method (AOAC, 2000), respectively. Neutral detergent fiber (NDF) and acid detergent fiber (ADF) were determined using a semi-automatic fiber analyzer (ANKOM 200i) according to the method of Van Soest (Van Soest et al., 1991).

The silage samples (20 g) were taken and homogenized with 180 mL of distilled water in a blender for 1 min and then filtered through 4 layers of gauze and qualitative filter paper. The pH value of the leachate was immediately determined with a portable pH meter (SX-620, Shanghai). The contents of lactic acid (LA), acetic acid (AA), propionic acid (PA), and butyric acid (BA) were determined by gas chromatography (model GC-6800, Beijing Beifen Tianpu Instrument Technology Co., Ltd.), and the chromatographic conditions were as follows: Φ6 mm × 2m quartz glass packed column (15% FFAP as stationary phase, 80–100 mesh Chromosorb as tensile strength), column temperature of 150°C, inlet temperature of 220°C. The column temperature was 150°C, the inlet temperature was 220°C, the injection volume was 1 μL, the FID detector temperature was 280°C, the carrier gas was high purity N_2_ with a flow rate of 30 mL/min, and a pressure of 200 kPa, the gas was H_2_ with a flow rate of 30 mL/min and the combustion gas was the air with a flow rate of 300 mL/min. The LA content was determined using high-performance liquid chromatography (Shimadzu LC-20A). Chromatographic conditions were as follows: the mobile phase was a solution of 3 mmol/L perchloric acid at a flow rate of 1 mL/min, the column temperature was 60°C, and the detector was an SPD-20A ultraviolet detector at a detection wavelength of 210 nm.

### 2.3 Determination of bacterial containment and aerobic stability

Another 20 g of silage was sampled and homogenized with 180 mL of sterilized normal saline and remained for 30 min to make a 10^1^ bacterial diluent, then 10^2^, 10^3^, 10^4^, 10^5^, and 10^6^ bacterial diluents were prepared sequentially in the aseptic operating table. LAB, yeast, and mold in silage were counted using the spread plate method with MRS agar medium (MRS) and potato dextrose agar (PDA), respectively. LAB was continuously cultured for 2 days in an anaerobic incubator at 37 °C, and yeast and mold were cultured continuously for 2–3 days at 30°C under aerobic conditions. Smooth, round white colonies on the medium were used for LAB counting; moist, smooth, opaque, large, and flat colonies on the medium were used for yeast counting; loose colonies with slender, fluffy, and flocculated mycelium on the medium were used for mold counting, and the unit of colony counting was expressed as log10 CFU/g.

After 14 days and 56 days of ensiling, samples (100 g) of each group were collected, respectively, and placed in a special polyethylene tank without sealing and compaction, but covered the mouth with 2 layers of gauze and a thermometer probe with a range of 50°C inserted into the center of the silage samples to determine the temperature. The temperature was recorded every 0.5 h and the recording was stopped when the silage temperature exceeded the ambient temperature by 2°C (that is, aerobic stability was reached).

### 2.4 Microflora analysis

#### 2.4.1 Extraction of total bacterial DNA

Approximately 5 g of fresh silage sample was placed in a 50 mL sterile tube, 25 mL of 0.1 M potassium phosphate buffer (pH = 8.0) was added, washed by ultrasonication for 1 min, and vortexed and oscillated for 10 s, this step was repeated twice. After washing, the samples were removed and filtered through four layers of sterile gauze. After combining the filtrates, the microbial precipitates were collected for DNA extraction by centrifugation at 4°C for 10 min using a centrifuge (Eppendorf 5424R, Germany) at 13,000 r/min (Bodenhausen et al., 2013). Three replicates were performed in each group. The total DNA of the microbial community was extracted according to the instructions of the EZNA^®^ Soil DNA kit (Omega Bio-tek, Norcross, GA, USA) and the quality of the DNA extracts was examined using 1% agar gel electrophoresis. DNA concentration and purity were determined using NanoDrop2000; 338F (5′-actcctacgggaggCAGCAGCAG-3′) and 806R (5′-GACTACHVGGGTWTCTAAT-3′) were used for the PCR of the variable region of the 16S rRNA gene V3-V4 of the 16S rRNA gene for PCR amplification. The amplification procedure was as follows: predenaturation at 95°C for 3 min, 27 cycles (denaturation at 95°C for 30 s, annealing at 55°C for 30 s, and extension at 72°C for 30 s), followed by a stable extension at 72°C for 10 min, and finally storage at 4°C (PCR instrument: ABI GeneAmp^®^ Model 9700). The PCR reaction was carried out in a 20 μL system, including 5 × FastPfu buffer 4 μL, 2.5 mM dNTP 2 μL, forward primer (5 uM) 0.8 μL, reverse primer (5 uM) 0.8 μL, TransStart FastPfu DNA polymerase 0.4 μL, 0.2 μL BSA, 10 ng template DNA and double steaming supplemented to 20 μL of each sample.

#### 2.4.2 PCR amplification and high-throughput sequencing of 16s rRNA genes

PCR products from the same samples were mixed and then the PCR products were recovered using a 2% agar gel. The recovered products were purified using the AxyPrep DNA Gel Extraction Kit (Axygen Biosciences, Union City, CA, USA). 2% agarose gel electrophoresis and a Quantus™ Fluorometer (Promega, USA) were used for the detection and quantification of recovered products. Libraries were constructed using the NEXTflexTM Rapid DNA Sequencing Kit (Bioo Scientific, USA) by (1) joint ligation; (2) removal of joint self-ligated segments using a magnetic bead sieve; (3) enrichment of library templates by PCR amplification; and (4) recycling of PCR products using magnetic beads to obtain the final library. Sequencing was performed using Illumina's Miseq PE300 platform (Shanghai Maggie's Biomedical Technology Co., Ltd.). The raw sequencing data of this study were available in the NCBI SRA database with the BioProject ID of PRJNA1053646.

### 2.5 Statistical analysis

The experimental data were summarized by Excel (2010) and statistically analyzed using SPSS version 19.0 for Windows (SPSS Inc., Chicago, IL, USA). One-way analysis of variance (ANOVA) and LSD methods were performed for statistical analysis and multiple comparisons.

## 3 Results

### 3.1 Nutrient composition, pH value, and bacterial count of alfalfa in sandy grasslands before ensiling

The mean pH value of fresh alfalfa sap was 7.02, and the nutrient composition of alfalfa before silage is shown in Table 1, where the numbers of LAB, yeast and mold are shown in Table 2.

**Table 1:** Nutrient composition and pH value of alfalfa in sandy grasslands before ensiling (g/kg DM).

**Item**	**Dry matter (fresh weight basis)**	**Crude protein**	**Ether extract**	**Water-soluble carbohydrates**	**Neutral detergent fiber**	**Acid detergent fiber**	**pH value**
Content	222.59 ± 2.31	206.86 ± 2.02	22.17 ± 1.47	41.62 ± 5.12	415.03 ± 5.19	373.7 ± 1.93	7.02 ± 0.003

**Table 2:** Number of LAB, yeast, and mold in alfalfa in sandy grasslands before ensiling (log10 CFU/g).

**Item**	**LAB**	**Yeast**	**Mold**
Content	5.54	5.61	5.58

### 3.2 Nutrient composition of alfalfa silage in sandy grasslands

As shown in Table 3, after 14 days of ensiling, the DM content of the alfalfa silage in group M and LM was extremely significantly higher than in group CK (*P* < 0.01), but the difference between group L and CK was not significant (*P* > 0.05). There were no significant differences in the CP content among the groups (*P* > 0.05). The EE content was extremely significantly higher in groups L, M, and LM than in group CK (*P* < 0.01). The WSC content was extremely significantly lower in groups L, M, and LM than in group CK (*P* < 0.01). The NDF and ADF content was extremely significantly lower in groups M and LM than in group CK (*P* < 0.01).

**Table 3:** Nutrient composition of alfalfa silage in sandy grasslands after 14 days and 56 days of ensiling (g/kg DM).

**Item**		**CK**	**L**	**M**	**LM**	**SEM**	***P*-value**
14 d	Dry matter (Fresh weight basis)	207.23B	210.20B	228.09A	234.25A	3.035	<0.001
	Crude protein	210.10	219.88	225.54	219.55	5.022	0.081
	Ether extract	23.89C	30.41B	34.03AB	37.45A	1.333	<0.001
	Water-soluble carbohydrates	94.40A	58.62C	71.39B	50.65D	1.623	<0.001
	Neutral detergent fiber	371.91A	370.56A	313.39B	302.50B	4.253	<0.001
	Acid detergent fiber	358.14A	357.54A	301.89B	294.65B	3.781	<0.001
56 d	Dry matter (Fresh weight basis)	206.90ab	203.68b	224.43ab	230.01a	7.914	0.026
	Crude protein	220.86	221.00	220.77	227.17	7.570	0.793
	Ether extract	28.07C	31.85BC	38.39AB	43.16A	2.136	<0.001
	Water-soluble carbohydrates	50.06C	109.68A	107.17A	77.41B	5.689	<0.001
	Neutral detergent fiber	409.49A	395.42B	290.38D	316.85C	3.676	<0.001
	Acid detergent fiber	398.68A	366.98B	283.03D	305.59C	6.356	<0.001

Different lowercase letters in the same row of data indicate significant differences (P <0.05), capital letters indicate extremely significant differences (P <0.01), and the same letter or no letter indicates nonsignificant differences (P > 0.05); SEM, standard error of the mean. CK, control group; L, Lactobacillus plantarum group; M, molasses group; LM, Lactobacillus plantarum +molasses mixture group. The following tables are the same.

After 56 days of ensiling, the alfalfa DM content was not significantly different among groups (P > 0.05) but was significantly higher in the LM group than in the M group (*P* < 0.05). There were no significant differences in the CP content among the groups (P > 0.05). However, the EE content was extremely significantly (*P* < 0.01) higher in groups M and LM than in group CK. The WSC content was extremely significantly higher in groups L, M, and LM than in group CK (*P* < 0.01). The content of NDF and ADF in all other groups were extremely significantly lower than in the CK group (*P* < 0.01).

### 3.3 Fermentation quality of alfalfa silage in sandy grasslands

As shown in Table 4, after 14 days of ensiling, the pH values in group L, M, and LM were extremely significantly lower than those in group CK (*P* < 0.01), while the difference in pH values between group M and LM was not significant (*P* > 0.05). The LA content of groups M and LM was extremely significantly higher than in groups CK and L (*P* < 0.01), while there was no significant difference in LA content between groups M and LM (P > 0.05). There was no significant difference in the content of AA and PA in all groups (*P* > 0.05), and BA was not detected in any of the groups.

**Table 4:** Fermentation quality of alfalfa silage in sandy grasslands after 14 days and 56 days of ensiling (g/kg DM).

**Item**		**CK**	**L**	**M**	**LM**	**SEM**	***P*-value**
14 d	pH value	6.46A	5.50B	5.12C	4.96C	0.059	<0.001
	Lactic acid	7.50C	10.76B	22.93A	21.77A	0.772	<0.001
	Acetic acid	2.68	3.12	2.81	2.82	0.234	0.956
	Propanoic acid	0.03	0.03	0.01	0.01	0.013	0.264
	Butyric acid	ND	ND	ND	ND	-	-
56 d	pH value	5.60A	4.37B	4.18C	4.09C	0.042	<0.001
	Lactic acid	9.88C	12.94B	25.05A	23.71A	0.749	<0.001
	Acetic acid	4.05B	6.17A	3.13B	3.60B	0.335	<0.001
	Propanoic acid	0.17a	0.13ab	0.01b	0.01b	0.047	0.016
	Butyric acid	ND	ND	ND	ND	-	-

ND indicates that no data has been detected. The following tables are the same.

After 56 days of ensiling, the pH values of group L, M, and LM were extremely significantly lower than that of group CK (*P* < 0.01), and the pH values were not significant between group M and LM (P > 0.05). While the LA content was extremely significantly higher in groups M and LM than in other groups (*P* < 0.01), and extremely significantly higher in group L than in group CK (*P* < 0.01), but there were no significant differences between groups M and LM (*P* > 0.05). The AA content was extremely significantly higher in group L than in other groups (*P* < 0.01), while there was no significant difference among groups CK, M, and LM (*P* > 0.05). The PA content was significantly higher in group CK than in groups M and LM. BA was not detected in any of the groups.

### 3.4 Bacterial count and aerobic stability of alfalfa silage in sandy grasslands

As shown in Table 5, after 14 days of ensiling, the bacterial count of LAB in groups from high to low was LM > M > L > CK (*P* < 0.01). And LAB in the LM group was extremely significantly higher than in other groups (*P* < 0.01), but the difference between the L and M groups was not significant (*P* > 0. 05). The yeast bacterial count in group L, M and LM were extremely significantly higher than in group CK (*P* < 0.01), but the difference between group L and LM was not significant (*P* > 0.05). Mold was detected in all groups. The aerobic stabilization time from long to short was L > LM > M > CK, respectively (*P* < 0.01).

**Table 5:** Bacteria count and aerobic stability of alfalfa silage in sandy grasslands after 14 days and 56 days of ensiling (log10 CFU/g).

**Item**		**CK**	**L**	**M**	**LM**	**SEM**	***P*-value**
14 d	*LAB*	6.83C	6.99BC	7.07B	7.33A	0.061	<0.001
	*Yeast*	6.91C	7.28A	7.10B	7.29A	0.045	<0.001
	*Mold*	1.33	1.33	3.33	1.83	-	-
	aerobic stability (h)	47C	120A	109B	115B	2.687	<0.001
56 d	*LAB*	6.88C	7.12B	7.36A	7.50A	0.048	<0.001
	*Yeast*	6.18A	5.62B	6.45A	5.70B	0.113	<0.001
	*Mold*	ND	ND	ND	1.49	-	-
	aerobic stability (h)	75C	120A	113B	117B	1.700	<0.001

After 56 days of ensiling, the bacterial count of LAB from high to low was LM > M > L > CK, respectively (*P* < 0.01). The yeast bacterial count in groups CK and M were extremely significantly higher than that in groups L and LM (*P* < 0.01). Mold was detected in LM group. The aerobic stabilization time from long to short was in group L > LM = M > CK (*P* < 0.01).

### 3.5 Microflora of alfalfa silage in sandy grasslands

#### 3.5.1 Alpha diversity of microflora

**Figure 1:**
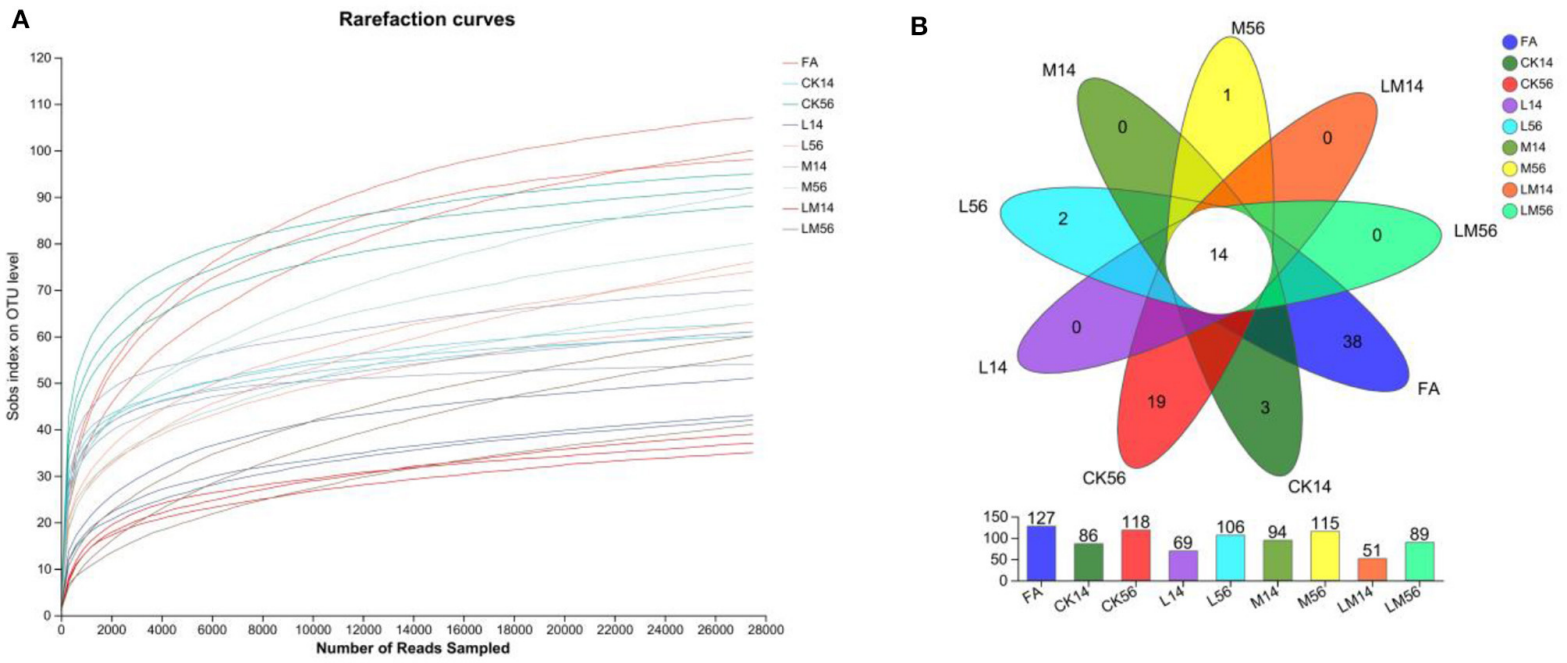
Dilution curves **(A)** and Venn diagrams of species **(B)**. FA, fresh alfalfa; CK14, control group for 14 days; CK56, control group for 56 days; L14, *Lactobacillus plantarum* group for 14 days, L56, *Lactobacillus plantarum* group for 56 days; M14, molasses group for 14 days, M56, molasses group for 56 days; LM14, *Lactobacillus plantarum*, and molasses mixture group for 14 days, LM56, *Lactobacillus plantarum*, and molasses mixture group for 56 days. The following figures are the same.

The OTU clustering analysis of the non-repeated sequences (excluding single sequences) of each group of colonies was performed according to 97% similarity, and a total of 1019726 valid sequences were obtained, with an average number of 37,767 valid sequences per sample, and an average length of 427 bp for each sequence. A total of 234 OTUs were obtained for 8 phyla, 14 classes, 46 orders, 76 families, 124 genera, 182 species, and a total of 234 OTUs. The dilution curves (Figure 1A) were close to smooth, indicating that the amount of data from this sequencing meets the analysis requirements. As shown in Figure 1B, the number of OTUs in groups M, L, and LM decreased compared to groups FA and CK, and the number of OTUs from more to less was FA > CK56 > M56 > L56 > M14 > LM56 > CK14 > L14 > LM14, of which the number of OTUs common to each group was 14, and the number of OTUs specific to FA, CK56, CK14, L56 and M56 was 38, 19, 3, 2, and 1, respectively.

As shown in Table 6, the species coverage of all tested samples was >99%, indicating that the sequencing results could accurately reflect the characteristics of the bacterial microbial communities.

**Table 6:** Bacterial alpha diversity of alfalfa silage in sandy grasslands after 14 days and 56 days of ensiling.

**Item**		**FA**	**CK**	**L**	**M**	**LM**	**SEM**	***P*-value**
14 d	ACE index	114.14a	82.17ab	80.85ab	92.93ab	56.39b	18.686	0.108
	Chao1 index	113.07A	72.31B	64.44B	71.00B	50.67B	6.871	<0.001
	Shannon index	2.23B	2.75A	1.48C	2.42AB	0.33D	0.156	<0.001
	Simpson index	0.19C	0.09D	0.33B	0.15CD	0.89A	0.026	<0.001
	Coverage (%)	99.94	99.96	99.96	99.97	99.96	0.011	0.120
56 d	ACE index	110.22	105.58	135.86	127.55	75.30	30.165	0.367
	Chao1 index	107.64a	106.65a	111.56a	109.40a	65.45b	15.694	0.068
	Shannon index	2.23B	3.11A	2.02B	1.99B	1.14C	0.203	<0.001
	Simpson index	0.19b	0.07c	0.21b	0.26b	0.43a	0.072	0.008
	Coverage (%)	99.95	99.95	99.92	99.92	99.94	0.014	0.092

After 14 days of ensiling, the FA ACE index was significantly higher than that of the LM group (*P* < 0.05), and the difference among groups CK, L, and M was not significant (*P* > 0.05). The Chao1 index of FA was extremely significantly higher than that of other groups (*P* < 0.01), and the difference among the other groups was not significant (*P* > 0.05). The Shannon index of the CK group was extremely significantly higher than that of the FA, L, and LM groups (*P* < 0.01), but not different from group M (*P* > 0.05). The Simpson index of the LM, L, and FA groups was extremely significantly higher than the CK group (*P* < 0.01).

After 56 days of ensiling, the differences in the ACE index among the groups were not significant (*P* > 0.05). The Chao1 index of the FA, CK, L, and M groups was not significantly different (*P* > 0.05), and all were significantly higher than the LM group (*P* < 0.05). The Shannon index of the CK group was extremely significantly higher than that of other groups (*P* < 0.01), and the differences among groups FA, L, and M were not significant (P > 0.05). The Simpson index was significantly higher in the LM group than in other groups (*P* < 0.05), and the differences were not significant among groups FA, L, and M (*P* > 0.05), which were significantly higher than the CK group (*P* < 0.05).

#### 3.5.2 Beta diversity of microflora

**Figure 2:**
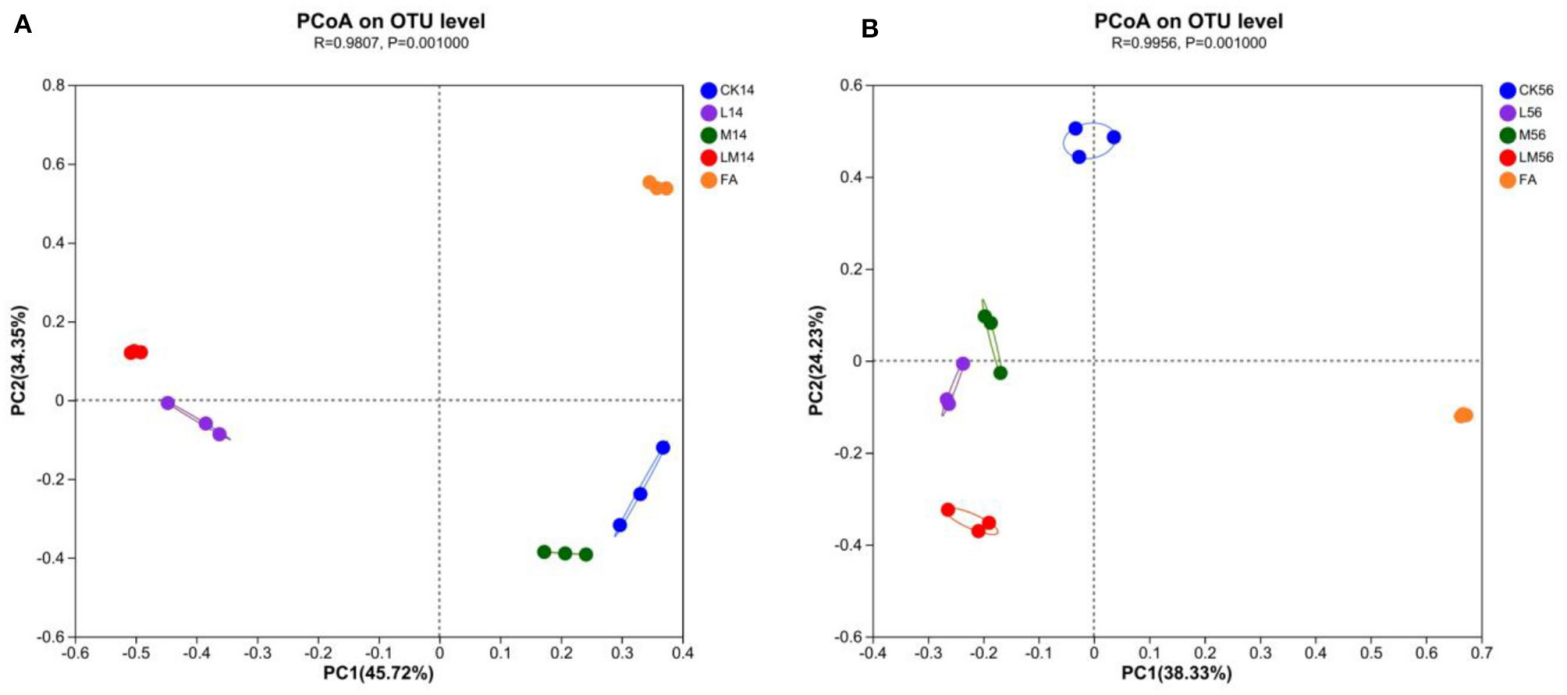
PCoA analysis of alfalfa silage in sandy grasslands after 14 days **(A)** and 56 days **(B)** of ensiling.

PCoA (principal coordinates analysis) was used to reveal similarities and differences in community composition among groups. After 14 days and 56 days of ensiling, all groups showed significant aggregation away from each other (Figures 2A, B), indicating that the bacterial communities differed significantly among the groups. As can be seen in the figures, as far as the bacterial communities were concerned, after 14 days of ensiling, group M was more similar to group CK, group LM was more similar to group L, and was more different from group CK, M and FA. After 56 days of ensiling, groups L and M were closer, but both were more different from groups LM, CK, and FA, with group LM being the most different from the other groups.

#### 3.5.3 Analysis of the relative abundance of microflora

**Figure 3:**
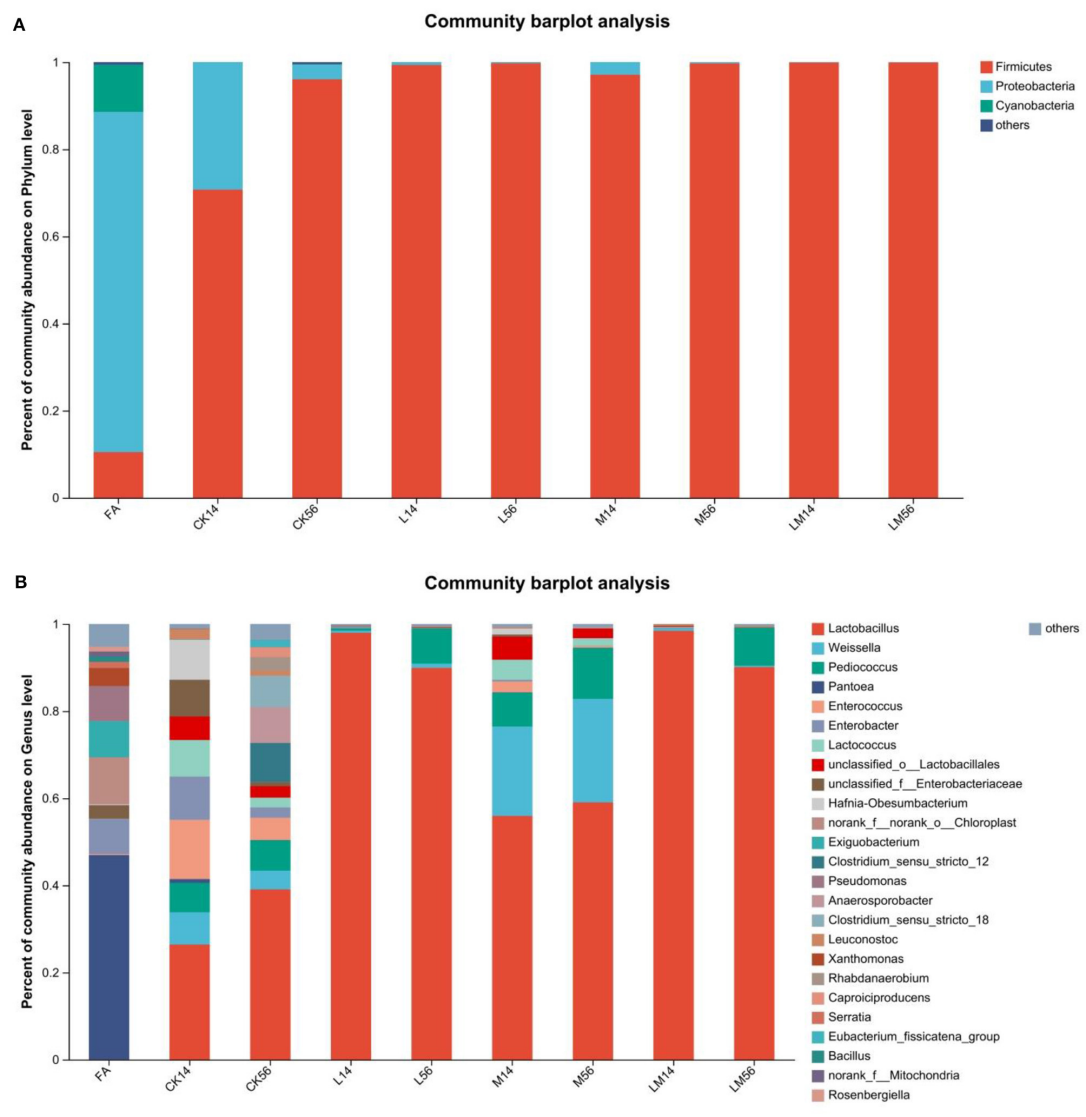
Bacterial composition of alfalfa silage in sandy grasslands after 14 days and 56 days of ensiling. Stacked bars indicate percent abundance. Bacterial communities are distributed at the phylum level **(A)** and genus level **(B)**. Small communities with abundances below 0.01 were merged with other communities.

As shown in Figure 3A, at the phylum level, in FA, the relative abundance of *Proteobacteria* was 78.07%. In groups CK, L, M, and LM, after 14 days of ensiling, the relative abundance of *Firmicutes* was 70.71%, 99.35%, 97.12%, and 99.88%, respectively. The relative abundance of *Proteobacteria* was 29.25%, 0.63%, 2.80%, and 0.10%, respectively. After 56 days of ensiling, the relative abundance of *Firmicutes* was 96.06%, 99.75%, 99.72%, and 99.89%, respectively. The relative abundance of *Proteobacteria* was 3.42%, 0.17%, 0.24%, and 0.06%, respectively.

As shown in Figure 3B, at the genus level, after 14 days of ensiling, *Pantoea* (46.91%) became the dominant bacteria in FA, followed by *Chloroplast* (10.81%), *Exiguobacterium* (8.27%), *Enterobacter* (8.09%), *Pseudomonas* (8.06%) and *Xanthomonas* (4.13%). In group CK, mainly contained *Lactobacillus* (26.4%), *Enterococcus* (13.59%), *Enterobacter* (9.94%), *Hafnia-Obesumbacterium* (9.22%), *unclassified_f_Enterobacteriaceae* (8.44%), *Lactococcus* (8.37%), *Weissella* (7.42%) and *Pediococcus* (6.74%). In group M, mainly contained *Lactobacillus* (55.94%), *Weissella* (20.51%), *Pediococcus* (7.76%), *unclassified_o_Lactobacillales* (5.27%), and *Lactococcus* (4.63%). The relative abundance of *Lactobacillus* in groups L and LM was 97.97% and 98.43%, respectively. After 56 days of ensiling, the relative abundance of *Lactobacillus* increased to 39.06% in the CK group, followed by *Clostridium_sensu_stricto_12* (8.95%), *Anaerosporobacter* (8.21%), *Clostridium_sensu_stricto_18* (7.23%), and *Pediococcus* (6.97%). In groups L, M, and LM, the relative abundance of *Lactobacillus* was 89.93%, 59.05%, and 90.11%, respectively. In groups L and LM, the relative abundance of *Pediococcus* was 8.07% and 8.75%, respectively. In group M, the relative abundances of *Weissella* and *Pediococcus* were 23.75% and 11.73%, respectively.

#### 3.5.4 LEfSe differential species analysis of microflora

**Figure 4:**
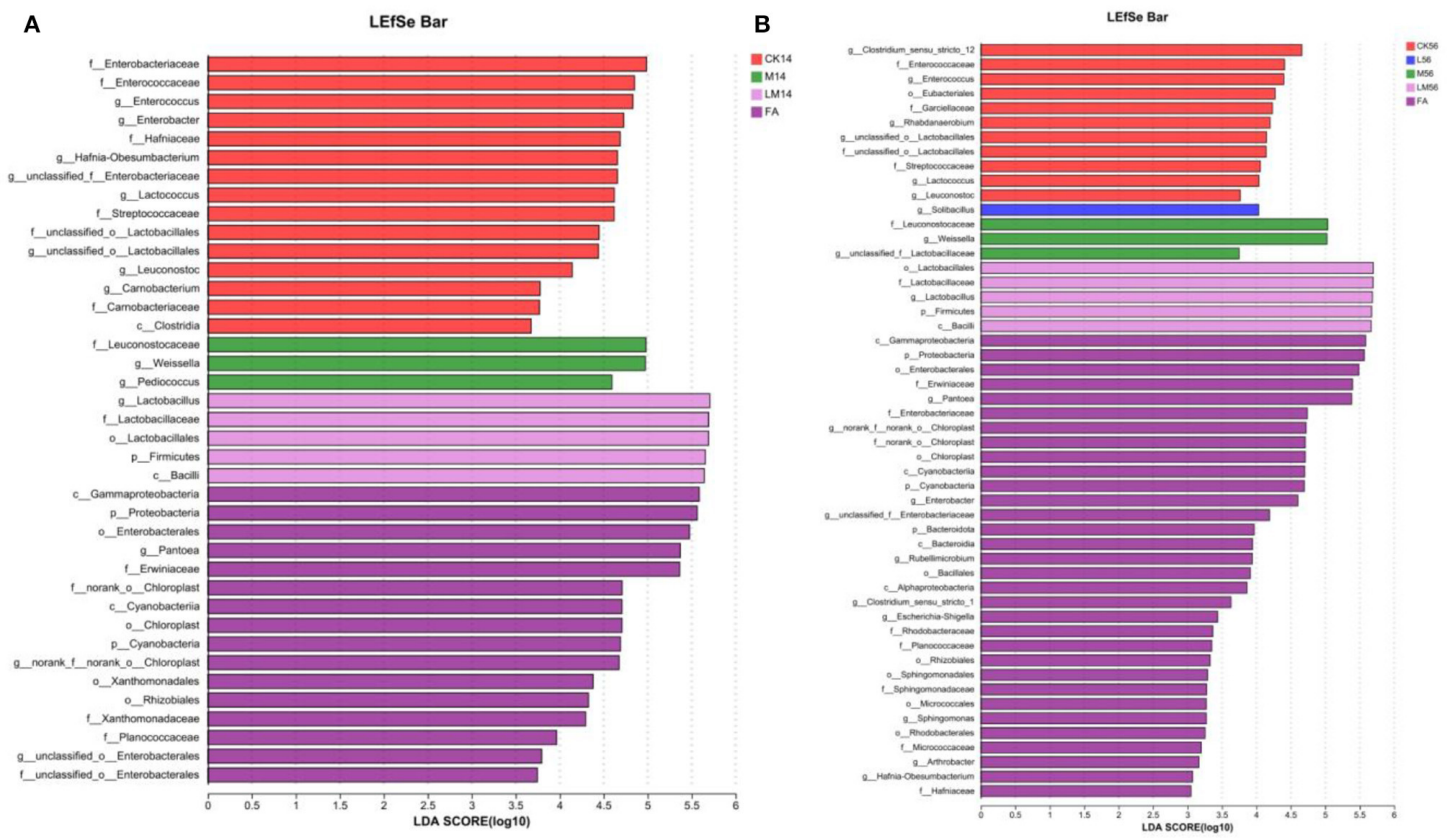
Alfalfa silage species differences in sandy grasslands after 14 days **(A)** and 56 days **(B)** of ensiling.

The differences in microflora species in alfalfa silage in sandy grasslands were analyzed using the multistage species discriminant analysis (LEfSe) (LDA = 3) (Figures 4A, B). As can be seen from Figure 4A, after 14 days of ensiling, species differences in FA (*c__Gammaproteobacteria, o__Enterobacterales*, etc.) were mostly *p__Proteobacteria*. Species differences in CK group (*f__Enterobacteriaceae, g__Enterococcus*, etc.) were mostly *p__Proteobacteria* and *p__Firmicutes*, and species differences in group M (*g__Pediococcus, g__Weissella, f__Leuconostocaceae*) and in group LM (*o__Lactobacillales, f__Lactobacillaceae, g__Lactobacillus*) were mostly *p__Firmicutes*.

As can be seen in Figure 4B, after 56 days of ensiling, species differences in group FA (*c__Gammaproteobacteria, o__Enterobacterales*, etc.) were mostly *p__Proteobacteria*. *In* group CK (*g__Clostridium_sensu_stricto_12, g__Enterococcus*, etc.) were mostly *g__Clostridium_sensu_stricto_12* and *p__Firmicutes*. *g__Solibacillus* was mostly in group L. Species differences in groups M and LM were similar to those of 14 days and were mostly *p__Firmicutes*.

### 3.6 Correlation of the composition of nutrients, the quality of fermentation, and the microflora of alfalfa silage in sandy grasslands

**Figure 5:**
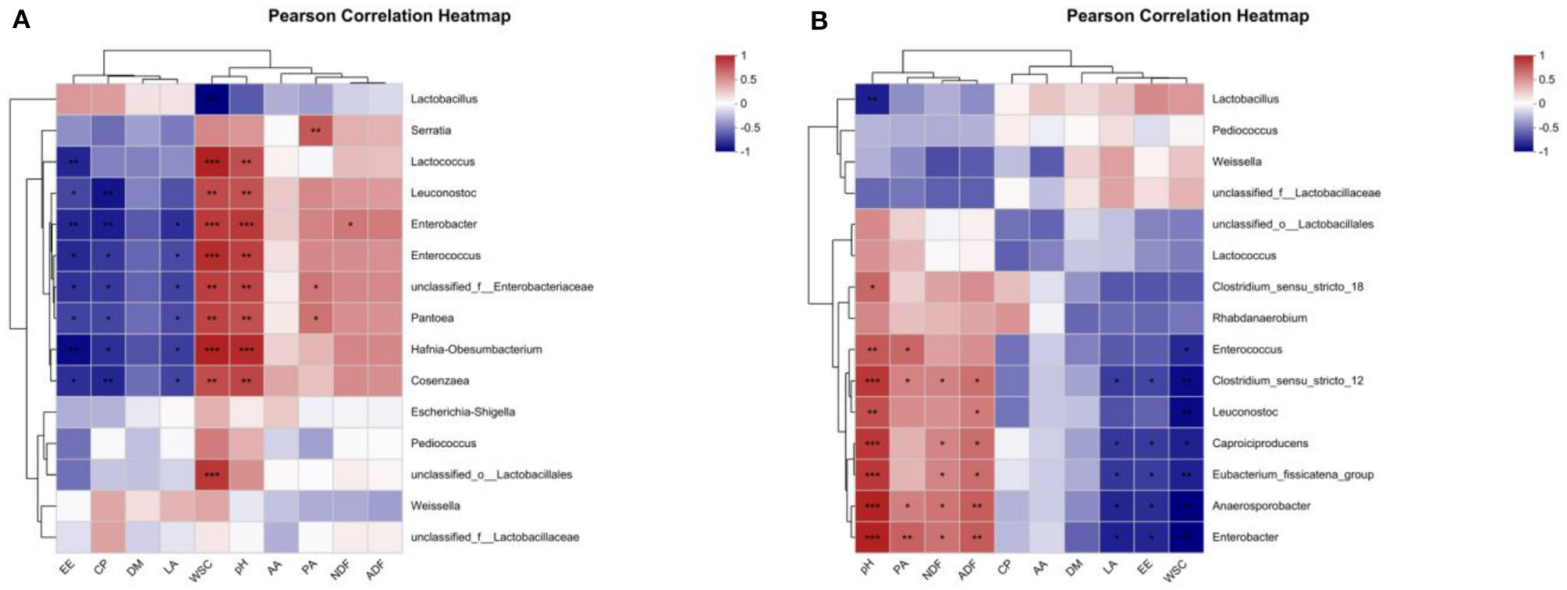
Heatmaps of Pearson's correlation among nutrient composition, fermentation quality, and microflora of alfalfa silage in sandy grasslands after 14 days **(A)** and 56 days **(B)** of ensiling. Heatmap colors indicate Spearman's correlation coefficient “R” (−1 ~ 1). r > 0 indicates positive correlation and *r* < 0 indicates negative correlation. *, ** and *** denote statistical significance levels of *P* < 0.05, <0.01 and <0.001, respectively.

A person correlation analysis was performed between nutrients and fermentation parameters with bacterial communities. As in the heatmap shown in Figure 5A, after 14 days of ensiling, the LA content was significantly negatively correlated (*P* < 0.05) with *Enterobacter, Hafnia-Obesumbacterium, unclassified_f_Enterobacteriaceae, Cosenzaea, Enterococcus*, and *Pantoea*. The WSC content was extremely significantly negatively correlated (*P* < 0.001) with *Lactobacillus* and extremely significantly positively correlated (*P* < 0.001) with Hafnia-Obesumbacterium, *Lactococcus, Enterococcus, unclassified_o_Lactobacillales, Enterobacter*, and was extremely significantly positively correlated with *unclassified_f_Enterobacteriaceae, Pantoea, Leuconostoc* and *Cosenzaea* (*P* < 0.01). The pH value was extremely significantly positively correlated (*P* < 0.001) with *Hafnia-Obesumbacterium, Enterobacter*, and significantly positively correlated (*P* < 0.001) with *Enterococcus, unclassified_f_Enterobacteriaceae, Cosenzaea, Lactococcus, Pantoea*, and *Leuconostoc* were extremely significantly positively correlated (*P* < 0.01).

As shown in Figure 5B, after 56 days of ensiling, the LA content was significantly negatively correlated with *Enterobacter, Anaerosporobacter, Caproiciproducens, Eubacterium_fissicatena_group, Clostridium_sensu_stricto_12* (*P* < 0.05). The WSC content showed a very significant negative correlation (*P* < 0.01) with *Enterobacter, Anaerosporobacter, Leuconostoc, Clostridium_sensu_stricto_12, Eubacterium_fissicatena_group*, and a significant negative correlation (*P* < 0.05) with *Caproiciproducens, Enterococcus*. The pH value was extremely significantly positively correlated (*P* < 0.001) with *Enterobacter, Anaerosporobacter, Caproiciproducens, Clostridium_sensu_stricto_12*, the *Eubacterium_fissicatena_group*, was significantly positively correlated with *Leuconostoc, Enterococcus*, and *Clostridium_sensu_stricto_18* (*P* < 0.05).

### 3.7 KEGG metabolic pathways of microflora in alfalfa silage in sandy grasslands

**Figure 6:**
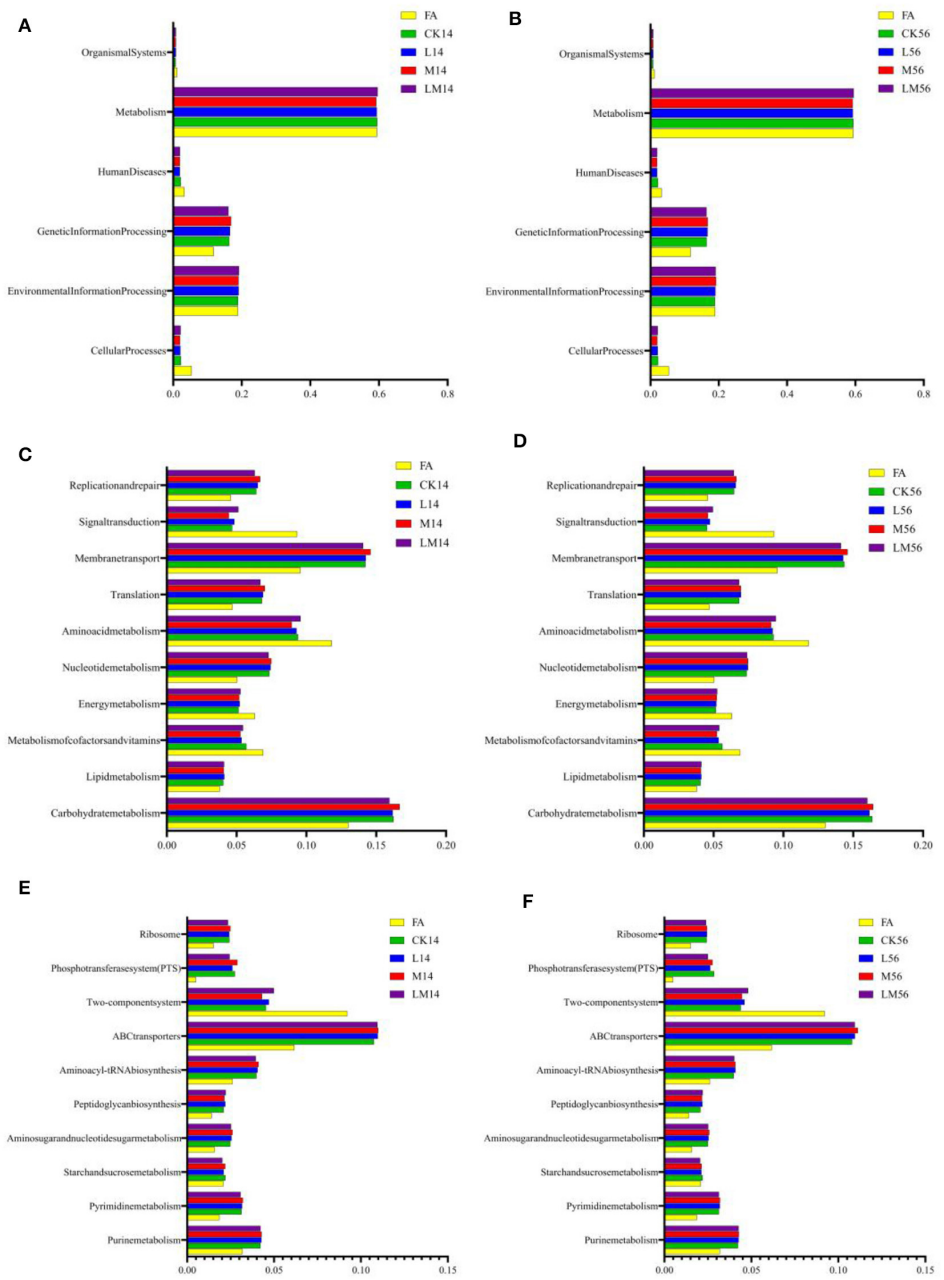
16S rRNA gene predictive function profiles of the KEGG metabolic pathway at level 1 [**(A)** after 14 days of ensiling; **(B)** after 56 days of ensiling], level 2 [**(C)** after 14 days of ensiling; **(D)** after 56 days of ensiling], and level 3 [**(E)** after 14 days of ensiling; **(F)** after 56 days of ensiling] in the different groups, as determined by Tax4Fun.

The 16S rRNA gene predictive function profiles at level 1 (Figures 5A, B), level 2 (Figures 6C, D), and level 3 (Figures 6E, F) of the pathways are illustrated in Figure 6. After 14 days and 56 days of ensiling, the metabolic pathways of metabolism, environmental information processing, and genetic information processing were significantly higher than other metabolic pathways (Figures 6A, B). Among them, carbohydrate metabolism, membrane transport, and amino acid metabolism were much higher than other metabolic pathways (Figures 6C, D). A more detailed comparison at the level of the third pathway showed that the metabolism of ABC transporters, the two-component system, purine metabolism, and aminoacyl-tRNA biosynthesis was significantly higher than the other metabolisms (Figures 6E, F). However, there were almost no significant differences in the metabolism of all groups at three levels of pathways after 14 days and 56 days of ensiling. But the carbohydrate metabolism and membrane transport in group M were extremely significantly higher than in other groups after 14 days of ensiling (*P* < 0.01).

## 4 Discussion

### 4.1 Effect of adding *Lactobacillus plantarum* and molasses on the nutrient composition of alfalfa silage in sandy grasslands

Studies have shown that the proliferation of undesirable microorganisms such as yeast, mold, and *Brucella* during silage is the main cause of DM loss (Wang et al., 2017). Compared to the CK group, the retention of DM was higher in the M and LM groups, suggesting that M and LM contributed to the reduction of the loss of DM from fermentation. On the contrary, the significant decrease in DM after the addition of L could be attributed to the addition of LAB to increase the intensity of fermentation, which promoted the decomposition of carbohydrates, and carbon dioxide that escaped within the conversion process to LA into AA and PA (Randby et al., 2012). This indicates that although the addition of L improved the fermentation speed and fermentation quality, it sacrificed a part of DM, and the final effect should be evaluated in combination with the content and digestibility of other nutrients in the silage. The CP content is an important indicator to determine the nutritional value of silage, which includes nitrogenous substances in the food as well as microbial proteins (MCP). In this experiment, the difference in CP content among the groups was not significant (*P* > 0.05), and even the addition of mycoprotein in group L did not cause a significant effect, indicating that L, M, and LM have less influence on the content of nitrogenous substances in the silage process, which is in line with the results of Zhu et al. (2022). The main reason for this is that the proteins in the raw material are preserved when the pH value is low enough to limit the activity of protein hydrolyzing bacteria (Luo et al., 2021). WSC is the main substrate for LAB fermentation, and during silage fermentation, it is consumed to produce LA, so WSC is the most important component for the production of high-quality silage (Silva et al., 2020; Ke et al., 2023). On the other hand, NDF and ADF are also important factors that influence animal feed intake and digestibility of silage (Li et al., 2019; Gül, 2023), and can be fermented by microorganisms to produce WSC or further fermented to produce organic acids. In this experiment, after 14 days of ensiling, L, M, and LM significantly reduced the content of WSC, NDF, and ADF, and increased the content of organic acids (LA) in alfalfa silage, indicating that the addition of *Lactobacillus plantarum* and molasses promoted the decomposition of carbohydrates and the production of organic acids. The microbial degradation of WSC, NDF, and ADF occurred almost simultaneously (Table 4). When compared in terms of WSC content, the degree of fermentation from fastest to slowest was LM > L > M > CK, and compared in terms of NDF and ADF content, the degree of fermentation from fastest to slowest was M > LM > L> CK. From this result, it is clear that the addition of *Lactobacillus plantarum* promotes the decomposition of nonstructural carbohydrates predominantly in the early stage of fermentation, whereas the addition of molasses promotes the decomposition of structural carbohydrates more effectively. According to the results of this experiment, the change in pH value (Table 4) was consistent with the WSC content (Table 3), and the lower WSC content was accompanied by a lower pH value, which, from this point of view, indicated that the addition of *Lactobacillus plantarum* (pH = 5.12) to promote the effect of early fermentation was better than molasses (pH = 5.50), while the best effect was achieved by adding the two at the same time (pH = 4.96). In terms of microflora, WSC was negatively correlated with *Lactobacillus plantarum* and positively correlated with *Enterococcus, Enterobacter, Pantoea*, and other undesirable fermentation-related microorganisms (Figure 5A), suggesting that the reduction of WSC and the resulting low pH values play an important role in the pre-silage period to inhibit the proliferation of undesirable microorganisms and improve the effect of silage.

After 56 days of ensiling, the content of NDF and ADF continued to decrease in groups L, M, and LM, but the content of WSC was extremely significantly higher than that of group CK (*P* < 0.01), indicating that at the later stages of fermentation, structural carbohydrates (NDF and ADF) were consumed mainly in group L, M and LM, but still maintained high organic acid production and low pH value, suggesting that carbohydrate fermentation at this time was still efficient and did not depend on fermenting WSC. From the changes in the bacterial genus shown in Figure 4, it can be seen that after 14 days of ensiling, the higher abundance of *g_Carnobacterium* in the CK group could promote the decomposition of NDF and ADF (Korsa et al., 2022), and after 56 days of ensiling, the increased abundance of *f_Lactobacillaceae* and *g_Clostridium_sensu_stricto_12* led to a large amount of consumption of WSC (Tang et al., 2017).

In this experiment, the proliferation of LAB during fermentation was promoted in groups L, M, and LM, and LAB-produced plasma lysozyme can degrade NDF and ADF (Korsa et al., 2022). Meanwhile, LAB promoted the conversion of fiber degradation products (such as WSC) to organic acids and lowered the pH value of silage, while a lower pH value can allow acid hydrolysis of digestible plant cells and further lead to increased digestion of NDF and ADF (Luo et al., 2021). Although the difference in NDF and ADF between group L and CK was not significant (*P* > 0.05) after 14 days of ensiling, it was extremely significantly lower than the CK group after 56 days of ensiling (*P* < 0.01). From microflora data, it is known that this may be due to *Weissella* proliferation after 56 days, which has genes to break down hemicellulose and cellulose (Hernández-Oaxaca et al., 2021).

After 14 days and 56 days of ensiling, the EE content of the M and LM groups was extremely significantly higher than the CK group (*P* < 0.01), and group L was extremely significantly higher than the CK group after 14 days of ensiling (*P* < 0.01), which may be attributed to fiber degradation or WSC, increasing the relative percentage of EE.

### 4.2 Effect of adding *Lactobacillus plantarum* and molasses on the quality of the fermentation of alfalfa silage in sandy grasslands

The pH value in the silage should be low enough to inhibit the multiplication of spoilage microorganisms and protein hydrolysis activity, thus reducing the loss of DM in the silage (Hattori et al., 1996). Insufficient acidification promotes the vigorous growth of *Clostridium butyricum* in the later stages of silage, which is the main cause of clostridial fermentation in alfalfa silage (Li et al., 2020). Studies have shown that the quality of silage fermentation is good when the pH value is 4.5 or lower (Hashemzadeh-Cigari et al., 2014). In this experiment, the pH values of alfalfa silage in groups L, M, and LM were significantly lower after 14 days and 56 days of ensiling and reached the ideal pH value of 4.5, where the pH values from low to high were LM <M <L, respectively, and the pH value of group CK (5.60) was extremely significantly higher than other groups and did not reach the ideal value (*P* < 0.01). This trend was already established at the early stage of 14 days, when the pH value of the CK group was 6.46, and the LM, M, and L groups had already decreased below 5.5, respectively. The pH value was negatively correlated with *Lactobacillus* and positively correlated with undesirable microorganisms such as *Enterobacter, Enterococcus*, and *Clostridium* (Figure 5); a lower pH value inhibited the proliferation of undesirable microorganisms. Therefore, inoculation with *Lactobacillu*s *plantarum* and the addition of molasses was very effective in reducing the pH value of the silage quickly and improving the quality of fermentation.

The production of organic acids is the main cause of the drop in the pH value in silage. Among them, LA is the ideal fermentation product in silage, produced mainly by WSC-consuming LAB, while AA, PA, and BA are less desirable (Besharati et al., 2022). According to the fermentation pattern of LAB, the fermentation products are mainly LA, while the products of mixed fermentation of multiple bacteria are mainly AA and other organic acids (Guo et al., 2018). In this experiment, the increase in LA content in groups L, M, and LM indicated that WSC was fully used, which was the main reason for the decrease in pH value, and the negative correlation between LA content and undesirable microorganisms, such as *Enterobacter, Enterococcus* and *clostridium* (Figure 5), indicated that LA could inhibit the proliferation of undesirable microorganisms. After 56 days of ensiling, the LA content of group M and LM was extremely significantly higher than that of group CK and L (*P* < 0.01), and the pH value of group M and LM was extremely significantly lower than that of group CK and L (*P* < 0.01), and the LA yield increased. Overall, although both *Lactobacillus plantarum* inoculation and molasses addition improved the fermentation quality of alfalfa silage, groups M and LM were more effective in controlling low pH values. Acetic acid (AA) is usually the second most concentrated organic acid in silage fermentation after lactic acid and has been proved to guarantee aerobic stability as an inhibitor of spoilage organisms (Danner et al., 2003). However, too much AA does not seem to be preferable, as too much acetic acid can reduce dry matter intake (DMI) of animals for palatability reasons. Studies have shown that an increase of 1 g acetic acid/kg DM led to a reduction of 1.2 g in DMI (per 100 kg body weight of animals) for acetic acid concentrations <17.3 g/kg DM (Gerlach et al., 2021). Therefore, the significant increase in AA content in group L caused by the addition of lactic acid bacteria does not seem to be the optimal result. PA is produced mainly by hetero-lactic bacteria, *Propionibacterium*, and *Enterobacter*, and is also degraded by citrate, malate, and amino acids (Tian et al., 2020). BA is produced primarily by fermentation of amino acids by *Clostridium butyricum*, leading to loss of nutrients and poor preservation, but BA is inhibited under well-fermented silage and lower pH value conditions (Krizsan and Randby, 2007). In this experiment, the PA content of groups L, M, and LM was kept at a lower level than that of group CK, indicating that a low pH value inhibits *Propionibacterium*. Meanwhile, the BA content was not detected in all treatment groups, indicating that alfalfa silage fermentation was good, with no increase in crude protein consumption (Table 3) and less acid production from poor fermentation.

### 4.3 Effect of *Lactobacillus plantarum* and molasses on bacterial count and aerobic stability of alfalfa silage in sandy grasslands

The maturity and type of silage fermentation are influenced by changes in the composition and abundance of the microbial community attached to the feed; in terms of metabolism, LAB are anaerobic, yeast is parthenogenetic anaerobic, and both have a similar cellular structure, yeast can degrade starch and sugar, while LAB degrades organic matter (Thomsen et al., 2007). Aerobic microorganisms, such as yeast, in the raw material dominate the early stages of ensiling and consume oxygen and some nutrients. When oxygen is reduced and conditions are suitable, LAB proliferates rapidly and secretes LA to inhibit the growth of aerobic microorganisms such as yeast and eventually become dominant (Ferrero et al., 2021; Okoye et al., 2023). However, this may be a kind of dynamic inhibition because yeast can still be isolated from sealed silage. Therefore, the presence of LAB and a lower pH value are necessary to inhibit yeast growth.

LAB is effective in lowering the pH value of silage reducing the population of yeast, and improving aerobic stability (Besharati et al., 2020a; Ferrero et al., 2021). In this experiment, the LM group had the lowest pH value after both 14 days and 56 days of ensiling, indicating that the LAB in this group had the highest intensity of activity and high aerobic stability, which is consistent with the results of aerobic stability (Table 5). After 56 days of ensiling, the pH values of group L, M, and LM were all <4.5, while group CK had not yet been reduced to <6.5. From the microflora point of view, there is a risk of undesirable fermentation mainly since the percentage of *Firmicutes* abundance in group CK is still low, and especially *Lactobacillus* are far from being overwhelmingly predominant to allow a rapid reduction of the pH value to the desired value. The same can be seen from the results of the bacterial count, which were extremely significantly lower (*P* < 0.01) in the CK and L groups than in the M and LM groups after 56 days of ensiling. At the same time, the yeast count was extremely significantly higher in the CK and M groups than in the LM group. Yeast can consume LA, leading to an increase in the pH value of the silage, making the silage more susceptible to deterioration due to aerobic activity (Thomas, 1991). Furthermore, the content of AA during silage fermentation should also be emphasized, and AA is often used as a poor silage fermentation inhibitor. Studies have shown that silage containing high concentrations of AA has lower yeast and mold populations and higher aerobic stability, AA can effectively reduce the pH value of silage, and its inhibitory effect on yeast is even greater than LA (Danner et al., 2003; Liu et al., 2020; Selwet, 2020; Yi et al., 2023), which is obvious in its inhibitory effect. In this experiment, although the difference in AA content was not significant in all groups after 14 days of ensiling (*P* > 0.05), the AA content of group L was extremely significantly higher than other groups after 56 days of ensiling (*P* < 0.01), while the yeast count of group L and LM was also extremely significantly lower than in group CK and M (*P* < 0.01). Furthermore, the L group with the highest AA content also had the highest aerobic stability, which confirms the bacteriostatic effect of AA, which is consistent with the results of Kim et al. (2016).

### 4.4 Effect of adding *Lactobacillus plantarum* and molasses on the microflora of alfalfa silage in sandy grasslands

The diversity and abundance of bacteria in the alfalfa silage was lower (*P* < 0.05) in group L and LM than in group CK and M after 14 days and 56 days of ensiling, which may be related to the addition of LAB so that they play a dominant role in the silage. This result is supported by several studies showing that the limited diversity of microbial in acidic environments is influenced by a decrease in the pH value (Kuang et al., 2013; Liu et al., 2020), and similar results were reported by Méndez-García et al. (2015). However, there was no decrease in the bacterial diversity of the silage in group M compared to group CK. This indicates that in the absence of exogenous LAB, the microflora did not change much in the early stage of ensiling when the WSC fermentation was carried out mainly. However, nonstructural carbohydrate fermentation was mainly carried out in the late stage, and significant changes in the microflora occurred. In summary, the richness of the microbial community in alfalfa silage was strongly influenced by the addition of exogenous LAB, whereas the diversity of silage microorganisms was strongly influenced by the addition of molasses or fermentation substrates.

*Firmicutes* and *Proteobacteria* are usually the dominant bacteria in alfalfa silage (McGarvey et al., 2013). *Firmicutes* are Gram-positive bacteria with a low G + C content in their genomes and degrade macromolecular compounds such as proteins, starch, and cellulose (Zeng et al., 2020). *Proteobacteria* are the largest bacterial phylum, including not only *Erwiniaceae, Sphingomonas Paucimobilis, Methylobacterium*, and *Pseudomonas adaceae*, but also pathogenic bacteria such as *Escherichia Coli, Vibrio*, and *Spirillum* (Santos et al., 2019). *Proteobacteria* play an important role in the degradation of organic matter and the carbon-nitrogen cycles during anaerobic digestion (Zhao et al., 2021). In this experiment, the structure of the bacterial community in alfalfa silage changed significantly with the fermentation time, the abundance of *Firmicutes* increased significantly and *Proteobacteria* decreased significantly in all groups from 14 days to 56 days of ensiling. After 56 days of ensiling, *Firmicutes* abundance was >99.72% in groups L, M, and LM, which was significantly higher than that in group CK and became the dominant phylum in groups L, M, and LM. This is related to the promotion of carbohydrate fermentation by the addition of *Lactobacillus plantarum* or molasses, and the dominant bacterial phyla of alfalfa silage with 3% molasses was reported to be *Firmicutes*, with an abundance of 95.58% (Luo et al., 2021). Molasses provides an additional fermentable substrate for LAB and promote the dominance of the silage bacterial community, thus shifting metabolism to LAB fermentation (Ni et al., 2017). Furthermore, the dominant bacteria in alfalfa silage supplemented with six different combinations of LAB were all *Firmicutes* with abundances ranging from 69.3% to 83.1%, followed by *Cyanobacteria* and *Proteobacteria* with abundances ranging from 3.9% to 26.4 and 0.4% to 14.6%, respectively (Wang et al., 2021). The results of the present study are consistent with the fact that a low pH value or anaerobic conditions during silage favor *Firmicutes* growth, which replaced *Proteobacteria* and were widespread after 60 days of silage (Keshri et al., 2018). The relative abundance of *Firmicutes* in LAB inoculated silage was higher at 15 and 30 days, but there was no difference after 60 days of ensiling (Zhao et al., 2021).

In this experiment, after 56 days of ensiling, the dominant bacteria in group M were *Lactobacillus, Weissella*, and *Pediococcus*, respectively, which all belong to *Lactobacillaceae* and predominantly use glucose, suggesting that the addition of molasses produced a promotional effect on them all. The relative abundance of *Weissella* was significantly higher than in other groups, indicating that *Weissella* was more sensitive to increasing glucose concentration, and, on the other hand, *Weissella* also had a great effect second only to LAB. This bacterium is visible in the digestive tract of animals and expresses β-glucosidase (Lee et al., 2012; Fusco et al., 2015), which facilitates the degradation of cellulose. In silage fermentation dominated by LAB, competing microorganisms will not be able to survive (Yang et al., 2019), and in this experiment, LAB had been promoted as the dominant genus in group L and LM, which inhibits the growth of other bacteria, and therefore explains a decrease in the abundance of the bacterial population. For example, the abundance of *Lactobacillus* in silage in groups L and LM was >97% on day 14 and decreased to 89% at 56 days. However, the abundance of *Lactobacillus* increased from 26.4% and 55.94% to 39.06% and 59.05% from 14 to 56 days in the group CK and M. LAB are the main producers of LA and play an important role in lowering the pH value and inhibiting mold growth in silage (Zhang et al., 2022). Conversion of LA to AA produces high concentrations of AA, which also lowers pH value and inhibits the growth of spoilage microorganisms (Tabacco et al., 2011). In this experiment, the highest LA concentration was found in group M, and the highest AA concentration was found in group L after 14 days and 56 days of ensiling, but among all groups of alfalfa silage, the best aerobic stability was found in group L. Although the combination of added *Lactobacillus plantarum* and molasses further optimized fermentation quality and nutritional value, *Lactobacillus plantarum* alone still provided excellent aerobic stability, which is consistent with the findings of Mu et al. (2021).

In general, metabolites produced by silage fermentation can affect the bacterial community and thus the quality of silage (Dong et al., 2020; Li P. et al., 2022). As shown in Figure 5, after 14 days of ensiling, the pH value and WSC content are significantly positively correlated with *Lactococcus, Enterobacter, Enterococcus*, and *Leuconostoc* (*P* < 0.01). *Lactobacillus* consumed a large amount of WSC to produce LA and decreased the pH value, which led to a very significant negative correlation between WSC and *Lactobacillus* (*P* < 0.001). And it is consistent with the results of Li et al. (2020). Low pH can inhibit the growth of *Enterobacter* and other harmful microorganisms during ensiling (Zhang et al., 2009; Yang et al., 2022). KEGG (Kyoto Encyclopedia of Genes and Genomes) has been used to explain the role of cells and organisms from a genomic perspective. During ensiling, various types of bacteria convert fermentable substrates to different metabolites through different metabolic pathways. Therefore, Tax4Fun was used to analyze the metabolic pathways of alfalfa silage in sandy grasslands based on KEGG. There were no significant differences in the metabolism of all groups at three levels of pathways after 14 days and 56 days of ensiling, indicating that the metabolism of the bacterial community stabilized after 14 days of ensiling. However, the carbohydrate metabolism and membrane transport in group M were numerically higher than in other groups after 14 days and 56 days of ensiling (Figures 6C, D), suggesting that adding molasses in silage may have the potential to enhances carbohydrate metabolism and membrane transport metabolism. This also explains the mechanism of LAB degrading WSC to produce LA and reduce the pH of silage.

## 5 Conclusions

In this study, the addition of L, M, and LM can improve the nutritional value of alfalfa silage in sandy grasslands, reduce the loss of DM, and increase the content of WSC. The contents of NDF, ADF, and EE were significantly decreased by M and LM. In addition, L, M, and LM had a good effect on increasing the number of LAB and inhibiting the number of yeasts, and L significantly increased the content of AA, which could improve the aerobic stability and extend the storage time, but may reduce the DMI of animals due to the palatability. In terms of the relative abundance of bacteria, L, M, and LM enabled *Firmicutes* to rapidly become the dominant phyla, with *Lactobacillus* having the highest abundance. According to Pearson's correlation, there is a very significant negative correlation between pH value and *Lactobacillus* (P <0.01) and a very significant positive correlation between pH value and *Lactococcus, Enterobacter, Enterococcus* and *Leuconostoc* (*P* < 0.01), which may be inhibited by *Lactobacillus* under the decreased pH value. Prediction of microbial genes showed that M could enhance the carbohydrate metabolism and membrane transport metabolism of alfalfa silage in sandy grasslands, which may contribute to LA production by LAB metabolism. In general, L, M, and LM all improved the fermentation quality and reduced the loss of nutrients to varying degrees, but considering the fermentation quality, the overall effects of M and LM were better than L. Therefore, M and LM are recommended to be used as silage additives in the process of alfalfa silage in sandy grasslands to improve the quality.

## Data availability statement

The datasets presented in this study can be found in online repositories. The names of the repository/repositories and accession number(s) can be found below: https://www.ncbi.nlm.nih.gov/, PRJNA1053646.

## Author contributions

WP: Writing – original draft, Writing – review & editing, Visualization, Methodology, Data curation. LZ: Writing – original draft, Visualization, Validation, Methodology, Investigation, Formal analysis, Data curation, Conceptualization. MW: Writing – review & editing, Visualization, Validation, Supervision, Project administration, Funding acquisition, Conceptualization. BW: Writing – review & editing, Supervision, Conceptualization. MX: Writing – review & editing, Methodology, Investigation, Formal Analysis, Data curation. RZ: Writing – review & editing, Formal analysis. JJ: Writing – review & editing, Investigation. CD: Writing – review & editing, Investigation. LD: Writing – review & editing, Investigation. YZ: Writing – review & editing, Resources. MB: Writing – review & editing, Software. HB: Writing – review & editing, Resources. XB: Writing – review & editing, Resources.
